# The utility of routine eeg in a general ICU

**DOI:** 10.1186/2197-425X-3-S1-A986

**Published:** 2015-10-01

**Authors:** I Rodini, M Papaioannou, D Kazis, M Konoglou, V Theodoridou, G Vassiliadou, M Bitzani

**Affiliations:** G. Papanikolaou Hospital, A' ICU, Thessaloniki, Greece; Aristotle University of Tessaloniki, 3rd Neurology Clinic, Thessaloniki, Greece

## Introduction

Recent recommendations of the European Society of Intensive Care Medicine suggest the use of continuous electroencephalography (cEEG) for the detection of nonconvulsive seizures (NCSz) and nonconvulsive status epilepticus (NCSE) not only for patients with primary acute brain injury, but also for patients with unexplained coma. However, cEEG monitoring is still unavailable in many ICUs and thus routine EEG is utilized for diagnostic purposes.

## Objectives

To estimate the diagnostic yield of routine EEG in a 15-bed adult general ICU.

## Methods

A retrospective study was performed that included all ICU patients who underwent at least one EEG over a 2 years period. Demographics, clinical data and EEG indication were collected from medical charts. All EEGs were reviewed by experienced neurophysiologists. Patterns were categorized as following: interictal epileptiform activity, clinical seizures with a clear EEG correlate, periodic discharges, slow polymorphic activity. Modifications in medical management after EEG were recorded.

## Results

Routine EEG was performed in 60 patients (39 males, 21 females) over the time period. The mean age was 52,1 (± 17,6) years, mean GCS: 5,7 (± 2,5) and mean Apache II score:20,3 (± 5,7). The overall mortality was 25%. All of them were mechanically ventilated. 43 patients were admitted due to acute central nervous system (CNS) disease and 17 patients due to sepsis and/or septic shock. The main indications for ordering a routine EEG were the unexplained impairment of consciousness (n = 34) and the presence of seizures or any other seizure-like movements (n = 26). Analysis of EEG findings showed: epileptiform activity = 6 (10%), clinical seizures with a clear EEG correlate = 2 (3,3%), periodic discharges = 9 (15%), slow polymorphic activity : 43 (71,7%). Periodic discharges pattern was further analyzed and 8 of them were characterized as NCSE according to certain criteria. All 8 NCSE patients had a CNS injury on admission and were on antiepileptic drugs (AED) on the EEG day. After EEG, the antiepileptic treatment was modified in 19 patients (31,6%).

## Conclusions

In our series, routine EEG was helpful in confirming NCSE among patients with brain injury and had an impact on management. However, exclusion of seizures in comatose ICU patients needs prolonged monitoring. The use of cEEG would allow us to identify NCSE more accurately and help to titrate AEDs.
